# The role of anesthesiologists in postpartum depression: current perspectives and future directions

**DOI:** 10.3389/fpsyt.2025.1511817

**Published:** 2025-03-19

**Authors:** Weijia Du, Xiaozhe Qian, Zhendong Xu, Zhiqiang Liu

**Affiliations:** ^1^ Department of Anesthesiology, Obstetrics and Gynecology Hospital of Fudan University, Shanghai, China; ^2^ Department of Thoracic Surgery, Renji Hospital, School of Medicine, Shanghai Jiaotong University, Shanghai, China; ^3^ Department of Anesthesiology, Shanghai Key Laboratory of Maternal Fetal Medicine, Shanghai Institute of Maternal-Fetal Medicine and Gynecologic Oncology, Shanghai First Maternity and Infant Hospital, School of Medicine, Tongji University, Shanghai, China

**Keywords:** postpartum depression, labor analgesia, obstetric anesthesia, cesarean section, anesthetic drugs

## Abstract

Postpartum depression is a common complication of childbirth that can seriously affect women, infants, and families. In 2020, the National Health Commission of the People’s Republic of China mandated depression and anxiety screenings during pregnancy and postpartum visits to ensure timely medical intervention and referrals to appropriate behavioral health resources. Anesthesiologists are indispensable members in obstetric practice. Optimal peripartum pain control can reduce stress response; promote breastfeeding; and lower maternal anxiety and depression. Recently, the discovery of the rapid and sustained antidepressant properties of ketamine and emerging evidence supporting the effectiveness of anesthetic drugs in the treatment of depressive disorders have positioned anesthesiologists on a new frontier for treating neuropsychiatric disorders. This review aimed to explore the impact of labor epidural analgesia, obstetric anesthesia, and anesthetic drugs on postpartum depression while discussing the emerging role of anesthesiologists in its prevention and treatment based on recent evidence.

## Introduction

Postpartum depression (PPD), a mood disorder experienced by women after childbirth, is associated with severe maternal and neonatal complications, such as suicide; infanticide; and developmental and behavioral problems in children and adolescents (1). Different diagnostic systems and clinical studies define PPD as that occurring within 4 weeks, 3 months, 6 months, or up to 1 year postpartum (2). No consensus exists on the exact duration of the postpartum period, with shorter time frames often used in biological studies and longer periods in sociological and intervention studies. Prevalence estimates for PPD vary widely due to differing criteria, the time frame considered, and geographical factors. A meta-analysis (3) of 565 studies from 80 countries and regions showed that the prevalence of PPD is approximately 17.22% worldwide, with Southern Africa having the highest prevalence rate at 39.96% (95% confidence interval [CI]: 27.81–83.48), whereas developed and high-income countries show significantly lower rates. Biological bases and sociological bases are two different etiologies of PPD. A subset of women who experience major depression only in the context of hormonal changes associated with late pregnancy and childbirth, while the higher PPD in Southern Africa might be due to sociological issues. Additional risk factors include history of depression, depression and anxiety during pregnancy, marital status, educational level, social support, spousal care, violent incidents, gestational age, breastfeeding, neonatal death, pregnancy planning, economic situation, life stress, smoking, alcoholism, and living environment. In China, the prevalence of PPD has increased in the last decade. A meta-analysis (4) of 95 studies from 23 regions in Mainland China revealed a PPD incidence of 14.8%, with higher rates in underdeveloped regions. Modifiable risk factors include education and income levels; anxiety; social support; poor health conditions; marriage and family relationships; and experiences of violence.

PPD is a significant public health issue triggered by various factors, requiring focused intervention by primary health care providers. Anesthesiologists are indispensable members in obstetrics practice. Optimal peripartum pain control can reduce stress response; promote breastfeeding; and lower maternal anxiety and depression. Recent discoveries of ketamine’s rapid and sustained antidepressant effects, along with emerging evidence of antidepressant properties in anesthetic drugs, have expanded the potential role of anesthesiologists. This review aimed to explore the impact of labor epidural analgesia (LEA), obstetric anesthesia, and anesthetic drugs on PPD while discussing the emerging role of anesthesiologists in its prevention and treatment based on recent evidence.

## Perinatal pain and PPD

In the non-obstetric population, the relationship between pain and depression is well-established (5), whereas the connection between perinatal pain and PPD remains unclear. Perinatal pain refers to pain occurring from 28 weeks of gestation to 1 week postpartum. Recently, although the association between perinatal pain and PPD has gained significant clinical research attention, its evidence has been inconsistent. Observational studies have shown that in susceptible women, pain at various perinatal stages (prenatal, labor, and postpartum) appears to be independently linked to depression scores at 6 weeks postpartum. Furthermore, labor and acute postpartum pain are associated with both acute and long-term PPD symptoms (6, 7). Acute postpartum pain can disrupt daily activities, mood, and sleep. Research has demonstrated that every 1-point (equivalent to 10 mm) increase in acute pain score after delivery correlates with an 8.3% increased risk of PPD at 8 weeks postpartum. A meta-analysis (8) of 19 studies involving 96,278 parturients found that perinatal pain significantly increases the risk of PPD (odds ratio [OR] = 1.43, 95% CI: 1.23–1.67). The underlying biological explanation for the relationship between pain and PPD may not solely involve tissue injury. A recent study has suggested that certain genetic loci (such as the rs 4633 C/C genotype) may be associated with both perinatal pain and PPD (9). Identifying common genetic components of these complex disorders may reveal relevant pathways or therapeutic targets and help develop risk stratification strategies for managing perinatal pain and PPD (10).

## Labor epidural analgesia and PPD

Based on these associations, mitigating labor pain may decrease the incidence of PPD. Continuous lumbar epidural analgesia, recognized as the gold standard for labor analgesia, has been the mainstay of neuraxial labor analgesia for several decades. Placement of an epidural catheter allows analgesia to be maintained until after delivery. Several studies have evaluated the association between LEA and PPD, yielding conflicting results.

### Results from observational and cohort studies

Observational studies are commonly conducted to investigate the association between LEA and PPD. Some studies have indicated that LEA can reduce the risk of PPD by approximately 70% at 42 days (11) and 46% at 2 years after childbirth (12). However, these studies faced misclassification bias, such as the absence of prenatal depression screening and inclusion of parturients with prenatal depression. A multicenter prospective cohort study (13) using propensity score matching demonstrated only a short-term (42 days postpartum) protective effect of LEA on PPD. Although the positive relationship between LEA and PPD has attracted broad attention, controversies remain. A population-based cohort study in Sweden failed to detect a statistically significant link between LEA and PPD at 6 weeks postpartum. However, prenatal depression and fear of childbirth emerged as risk factors for PPD (14). A Canadian longitudinal cohort study (15) found no correlation between LEA and PPD either pre-pregnancy or at 3, 6, and 12 months postpartum, with only the prenatal Edinburgh Postnatal Depression Scale (EPDS) score demonstrating predictive value of PPD. Recently, a prospective cohort study (16) in China has revealed similar results, showing that LEA did not reduce the incidence of PPD at 3 months postpartum. Factors such as chronic pain, prenatal EPDS score, family presence during delivery, and pain score 1 day postpartum were significantly related to PPD occurrence.

The inconsistency of the above findings may stem from limitations inherent in observational studies, which are prone to information bias and residual confounding. The presence of confounding factors can distort results; thereby, affecting their accuracy and reliability. First, the timing of PPD diagnosis is not uniform, with various studies defining PPD as an affective disorder occurring several days, weeks, months, or up to 1 year postpartum. Studies have found that PPD symptoms may persist up to 2 years after delivery (17). Some parturients showed no significant symptoms at 2 months postpartum, only to be diagnosed with PPD later. Future studies should include long-term follow-up to accurately assess the effectiveness of interventions. Second, although the relationship between LEA and PPD may reach statistical significance, the strength of this association is weak, with odds ratios below 4 (11–18). Weak associations are common in observational studies and may imply bias rather than causality (19). Third, differences in PPD diagnosis may cause over- or under-estimation of the effects of LEA on PPD. Most studies used the EPDS scale for PPD screening, with some applying an EPDS score of ≥12 to predict PPD risk^[14-20-21]^, whereas earlier studies used a diagnostic threshold of EPDS ≥9 (20, 21). However, EPDS is approximately 80% sensitive for diagnosing depression (22) and is designed for screening rather than establishing diagnosis. The structured interview based on the Manual of Mental Disorders-5 (DSM-5), such as the Mini-International Neuropsychiatric Interview, should remain the gold standard for diagnosing PPD, ideally conducted by psychiatrists or trained professionals. Moreover, because most data in existing studies are derived from medical records, potential confounding diagnoses might be missed due to incomplete collection of the patient’s medical history during clinical encounters. For instance, physical or mental problems may have an impact on labor analgesia or the onset of PPD.

### Results from randomized controlled trials

Randomized controlled trials (RCTs) are considered the gold standard for clinical research to evaluate the efficacy of therapies or interventions intended to improve outcomes. Compared to observational studies, RCTs are better equipped to control information, selection, and confounding biases, which may influence the risk of PPD. However, due to ethical issues, only two relevant RCTs have been conducted, to date. A study from India (23) found that the incidence of PPD at 6 weeks postpartum was similar between parturients receiving LEA and the control group (27.7% vs. 16.9%, *p* = 0.103). An analysis of all risk factors revealed that mental stress during pregnancy alone was an independent risk factor for PPD (adjusted OR = 11.17, 95% CI: 2.86–43.55). However, this study was limited by a small sample size. A study conducted in Singapore (24) randomized 773 participants to receive LEA or other modes of labor analgesia (nitrous oxide, meperidine, and remifentanil). The incidence of PPD at 6–10 weeks postpartum was similar between the two groups (15.9% vs.16.9%, *p* = 0.79). The authors suggested that the quality of labor pain control may have a more significant impact on PPD risk compared to the specific type of analgesic used (25).

### Results from meta-analyses

Meta-analyses sit at the top of the evidence-based medicine hierarchy. Almeida et al. (26) conducted an analysis of nine studies involving 4,442 parturients, showing no significant association between LEA and PPD (OR = 1.02, 95% CI: 0.62–1.66). Kountanis et al. (27) included 11 observational studies with 85,928 parturients and found no reduction in PPD risk with LEA (OR = 1.03, 95% CI: 0.77–1.37). Although these meta-analyses were not based on RCTs, the potential influence of unidentified confounders and biases in the available literature cannot be ignored, and the interactions among them may have influenced the results. Heterogeneity is another concern, as each study was adjusted for different confounding factors when testing the relationship between LEA and PPD. For instance, Ding et al. (11) adjusted for satisfaction with health services and participation in childbirth classes, whereas Orbach-Zinger et al. (28) controlled for marital status. Given the numerous risk factors for PPD, controlling for all known and unknown factors in each study is unfeasible, which can introduce confounding resulting in bias. An important source of bias is antenatal depression, which was not assessed in most studies except for two. Nahirney et al. (29) excluded women with an EPDS score of ≥13 at delivery, whereas Orbach-Zinger et al. (28) excluded women taking antidepressants during pregnancy, though they did not assess pre-delivery depression. Antenatal depression has been identified as a key predictor of PPD (15–26, 30), and women with depression may be more likely to request epidural analgesia (29). Thus, early screening may identify patients who are at high risk of developing PPD and provide an opportunity for consultation with the anesthesiologist to discuss labor analgesic plans.

The relationship between PPD and LEA is complex. The available evidence does not demonstrate a clear association between LEA and PPD. However, effective pain relief may have a greater influence on the risk of PPD than may have the mode of labor analgesia. A retrospective cohort study found that the degree of pain improvement predicted PPD. However, the predictive value was limited, with 45% pain improvement only decreasing the EPDS score by 1 point (25). Additionally, unmatched expectations for labor analgesia may cause maternal negative emotions, resulting in postpartum mood disturbances. An observational study from Israel showed that parturients who did not intend to use but eventually received LEA had a 50% increased risk of developing PPD (28). Regarding the medical costs, LEA might be preferred by high income mothers. Since economic status is related to the quality of support during labor and research has suggested a higher prevalence of PPD in low-income and middle-income countries compared to high-income countries (31). Future investigation evaluating the association between PPD and receiving LEA should discuss the economic status of mother which maybe a confounding factor.

## Obstetric anesthesia and PPD

Cesarean section is the most commonly performed abdominal surgery worldwide. However, women face dual challenges after Cesarean delivery (CD) for being post-partum and post-operative (32). The stress response, pain, and delayed lactation caused by surgical trauma can lead to negative emotions in postpartum women; General anesthesia (GA) increases the risk of persistent postoperative pain, affects postpartum recovery, delays lactation, and maternal infant contact, all of which are risk factors for PPD (33). However, there is currently disagreement on whether cesarean section and GA are associated with PPD.

### Cesarean vs. vaginal delivery

Although the potential correlation between CD and PPD has been studied, whether a direct link exists remains debatable. Some studies have found no significant association between CD and PPD. Lim et al. (6) suggested that PPD is more closely related to perinatal pain than is the mode of delivery, indicating that pain plays a major, independent role in persistent pain and depression. A multicenter longitudinal study (34) involving 1,288 women found that those with severe acute postpartum pain had a 2.5-fold increased risk of persistent pain and a 3.0-fold increased risk of PPD compared to patients with mild postpartum pain, independent of the type of delivery. These observations suggest that anesthesiologists and other obstetric care providers should place greater emphasis on managing acute pain during the first few days following childbirth to prevent long-term morbidities and improve outcomes in women after both CD and vaginal delivery.

In contrast, four meta-analyses (35–39) have suggested that CD is associated with increased odds of PPD compared to vaginal delivery. This association may stem from several factors, such as lower cortisol levels (40); reduced prolactin levels; increased interleukin-6 levels (41); bleeding; inflammation; stress response (42); and delayed first bonding with the newborn and breastfeeding initiation (43), all of which are PPD risk factors. A recent umbrella review (37), including 185 observational studies from 11 systematic reviews, has shown a relatively weaker association between CD and PPD (OR: 1.29) compared to other robust risk factors, such as premenstrual syndrome (OR: 2.20), violent experiences (OR: 2.07), and unintended pregnancy (OR: 1.53), which doubles the risk of PPD. The delivery mode may have a weak impact on the development of PPD but may interact with some risk factors, such as stress reactions, postoperative complications, breastfeeding difficulties, and unintended birth experiences, to increase the risk for PPD. Further studies are warranted to explore these interactive effects of CD alongside various moderating variables.

### General vs. neuraxial anesthesia

GA can delay maternal infant contact and lactation, increase postoperative pain, and reduce maternal satisfaction, all of which are risk factors for developing PPD (33). Guglielminotti and Li (44), in a study involving 428,204 parturients undergoing CD, showed that GA increased the risk of PPD by 54% (OR = 1.54, 95% CI: 1.21–1.95) and suicidal ideation or self-inflicted injury by 91% (OR = 1.91, 95% CI: 1.12–3.25) within 1 year after delivery. The increased risk of GA may result from delayed skin-to-skin contact for the maternal-infant dyad, prolonged breastfeeding initiation, increased postoperative pain, and reduced maternal satisfaction (45). Chen et al. (46) found that women who delivered under GA had a significantly higher risk of PPD within 90 days (hazard ratio [HR] = 1.71; 95% CI: 1.05–2.79) compared to those who delivered under neuraxial anesthesia. However, this increased risk was not observed within 180 days or 1 year post-delivery, possibly due to variations in study design and outcomes definitions. Hung et al. (47) reported a 139% increase in PPD risk with GA (OR = 2.393, 95% CI: 2.314–2.474) and a 74% increase with neuraxial anesthesia (OR = 1.74, 95% CI: 1.73–1.76) compared to normal spontaneous delivery. Women who underwent CD with GA were more likely to require antidepressants for sleep problems than were those who had neuraxial anesthesia. Moreover, emergency CD is at least 70% more prevalent among GA than among neuraxial anesthesia (44). GA was employed for emergency CD instead of neuraxial anesthesia for women with severe maternal morbidity, such as a major hemorrhage or abnormal placentation, which could cause confounding effects. Although these studies suggest a link between GA and PPD, they are limited by their observational design, which establishes associations rather than causality. If confirmed, these findings may have important influences on obstetric anesthesia practice, underscoring the need for reducing GA use during CD and providing mental health screening, counseling, and follow-up for women receiving GA. Further studies are warranted to investigate the association among the drugs and the procedures i.e., intravenous or inhalant, used in GA.

## Anesthetic drugs and PPD

Antidepressant medication is the most common treatment for PPD. Common pharmacotherapy for PPD includes serotonergic-based antidepressants and zuranolone. The discovery of the rapid antidepressant effects of ketamine and emerging evidence supporting the effectiveness of anesthetic drugs in treating depressive disorders have positioned anesthesiologists at the forefront of neuropsychiatric care (48). The discovery of the rapid and sustained antidepressant properties of ketamine, esketamine, and possibly dexmedetomidine has led to the development of new treatments of PPD.

### Ketamine and esketamine

In women undergoing CD, ketamine and esketamine have been used for GA induction and as adjuncts to neuraxial anesthesia, representing novel pharmacological treatments of depressive disorders (49). Given the similarities between PPD and other depressive disorders, these drugs may theoretically have similar effects on PPD. Ketamine, an N-methyl-d-aspartate receptor (NMDAR) antagonist, plays a pivotal role in managing depressive disorders. A meta-analysis (50) revealed that perioperative intravenous ketamine significantly reduced EPDS scores and the prevalence of PPD within 1 week postpartum, whereas the PPD score after 4 weeks postpartum showed no superiority. Esketamine, the S-enantiomer of ketamine, has approximately twice the affinity for NMDAR and an analgesic efficacy of 1.5 to 2 times greater than that of ketamine (51). Wang et al. (52) conducted a RCT and found that a single low dose of intravenous esketamine (0.2 mg/kg) administered after childbirth significantly reduced major depressive episodes at 42 days postpartum by approximately three-quarters, indicating the potential role of esketamine in treating PPD. Chen et al. (53) also conducted a RCT in women without prenatal depression and observed that intravenous esketamine (0.25 mg/kg) administered immediately after childbirth, followed by 50 mg of esketamine as an adjuvant in patient-controlled intravenous analgesia for 48 h after CD, improved depression symptoms and EPDS scores within 7 days after delivery. However, the effect diminished by postpartum days 14, 28, and 42. A recent meta-analysis (54) including 12 RCTs and two retrospective cohorts has shown significant effects of intravenous esketamine on reducing PPD incidence and EPDS severity scores during the first week and 42 days after delivery, indicating both short- and long-term antidepressant effects of esketamine.

Current findings support that esketamine and ketamine hold potential for preventing and treating PPD. However, the optimal timing, dosage, and administration methods for achieving the best efficacy remain to be clarified. Discrepancies in outcomes may stem from variations in these factors. Moreover, ketamine and esketamine are associated with significant neurologic and mental symptoms. Xu et al. (55) found that the incidence of transient neurologic or mental symptoms was 97.7% in patients undergoing CD who received intravenous esketamine (0.25 mg/kg) before incision. Although these adverse effects were transient and self-limiting, they required careful monitoring. Importantly, no drug-related serious adverse events were observed within 3 days after surgery.

### Dexmedetomidine

Dexmedetomidine, a highly selective α2-adrenoceptor (α2-AR) agonist, is commonly used in the perioperative period. Postmortem studies of individuals with depression who died by suicide have revealed increased α2-AR expression in multiple brain regions, and elevated platelet α2-AR density has been observed in patients with PPD (56). Preclinical experiments have also demonstrated that dexmedetomidine significantly improves depression-like behavior in sleep-deprived mice. Alterations in the α2-AR gene are associated with susceptibility to PPD (57). Many studies have demonstrated that dexmedetomidine can upregulate brain-derived neurotrophic factor (BDNF), a molecule closely associated with the pathogenesis and prognosis of PPD. The rapid antidepressant-like effects of ketamine are dependent on the rapid synthesis of BDNF (58). Dexmedetomidine may offer significant promise in modulating pathophysiological changes in depression and treating PPD due to its impact on α2-AR and BDNF levels. Recently, Zhou et al. (59) showed a significantly lower incidence of positive PPD screen (EDPS >9) at 7 and 42 days postpartum in the dexmedetomidine group compared to the control group (12.6% vs. 32.1% at day 7; HR = 0.39 [95% CI: 0.25–0.62]; *p* <.001; 11.4% vs. 30.3% at day 42, HR = 0.38 [95% CI: 0.23–0.61]; *p* < 0.001). Postpartum plasma BDNF levels were significantly higher in the dexmedetomidine group than that in the control group. In this RCT, the prophylactic administration of dexmedetomidine in the early postpartum period reduced the incidence of positive PPD screening, reduced postoperative pain, improved sleep quality, and maintained good safety. The antidepressant effect of dexmedetomidine may be related to its impact on pro-BDNF levels. A meta-analysis (60) of 13 RCTs showed that dexmedetomidine significantly improves the risk of PPD within 1 week postpartum. Multivariate regression analysis suggested that parturients aged < 30 years are more sensitive to dexmedetomidine and experience better therapeutic effects. Moreover, continuous background infusion combined with patient-controlled administration was superior to a single bolus injection; the therapeutic effect was better when the total dexmedetomidine infusion dose was ≤2 μg/kg. Although these preclinical and clinical findings suggest a promising application of dexmedetomidine to treating PPD, most studies are still preliminary. Further multicenter RCTs are needed to validate these findings and confirm dexmedetomidine’s effectiveness.

## Conclusion

PPD is a health issue that affects up to 1 in 7 women after childbirth in China and can significantly impact maternal functioning, bonding with the infant, family dynamics, and long-term emotional and cognitive development of infants. This review can have important implications for obstetric anesthesia practice, maternal health, and health care policy. First, optimal management of acute postpartum pain and reducing the short- and long-term sequelae of pain are crucial for preventing maternal and neonatal morbidities and improving their outcomes, not only in women after CD, but also in those after vaginal delivery. Clinicians should be aware that women who do not receive optimal pain management are at increased risk for PPD and early screening for this at-risk population is crucial for timely intervention. Second, more attention should be paid to women who undergo CD under GA during the early postpartum period. This population is likely to benefit from mental health screening, counseling, and other follow-up services. Third, the short-term antidepressant effects of ketamine and esketamine after CD were supported by the current research results with moderate certainty of evidence, but the long-term sustainability of treatment response is still uncertain. The neurologic and mental symptoms provoked by ketamine and esketamine might produce unfavorable experience and low patient satisfaction. Therefore, indications and the optimal dose of both drugs in this patient population need to be further clarified.
